# Insights into the Mechanism of Action of the Degraded Limonoid Prieurianin

**DOI:** 10.3390/ijms25073597

**Published:** 2024-03-22

**Authors:** Gérard Vergoten, Christian Bailly

**Affiliations:** 1 U1286—INFINITE, Lille Inflammation Research International Center, Institut de Chimie Pharmaceutique Albert Lespagnol (ICPAL), Faculté de Pharmacie, University of Lille, 3 Rue du Professeur Laguesse, 59006 Lille, France; 2 CNRS, Inserm, CHU Lille, UMR9020-U1277-CANTHER—Cancer Heterogeneity Plasticity and Resistance to Therapies, OncoLille Institut, University of Lille, 59000 Lille, France; 3 Institute of Pharmaceutical Chemistry Albert Lespagnol (ICPAL), Faculty of Pharmacy, University of Lille, 59006 Lille, France; 4 OncoWitan, Scientific Consulting Office, 59290 Lille, France

**Keywords:** dregeanin, fraxinellone, Hsp47, limonoids, prieurianin, *Trichilia prieuriana*, *Trichilia* species

## Abstract

Limonoids are extremely diversified in plants, with many categories of products bearing an intact, rearranged or fragmented oxygenated scaffold. A specific subgroup of fragmented or degraded limonoids derives from the tetranortriterpenoid prieurianin, initially isolated from the tree *Trichilia prieuriana* but also found in other plants of the Meliaceae family, including the more abundant species *Aphanamixis polystachya*. Prieurianin-type limonoids include about seventy compounds, among which are dregeanin and rohitukin. Prieurianin and analogs exhibit insecticidal, antimicrobial, antiadipogenic and/or antiparasitic properties but their mechanism of action remains ill-defined at present. Previous studies have shown that prieurianin, initially known as endosidin 1, stabilizes the actin cytoskeleton in plant and mammalian cells via the modulation of the architecture and dynamic of the actin network, most likely via interference with actin-binding proteins. A new mechanistic hypothesis is advanced here based on the recent discovery of the targeting of the chaperone protein Hsp47 by the fragmented limonoid fraxinellone. Molecular modeling suggested that prieurianin and, to a lesser extent dregeanin, can form very stable complexes with Hsp47 at the protein–collagen interface. Hsp-binding may account for the insecticidal action of the product. The present review draws up a new mechanistic portrait of prieurianin and provides an overview of the pharmacological properties of this atypical limonoid and its chemical family.

## 1. Introduction

Limonoids are highly oxygenated modified triterpenoids well represented in plants. They are largely present in the Meliaceae family and designated meliacins. They are also frequently encountered in Rutaceae and less frequently in Cneoraceae [1,2,3]. The tetranortriterpenoid limonin was the first limonoid identified as the bitter constituent of citrus fruits in 1841 [4,5]. Citrus, oranges, lemons and grapefruits contain limonin (**1**) and other bioactive limonoids such as nomilin (**2**) and obacunone (**3**), endowed with antioxidative, anti-inflammatory, antimicrobial, antiviral, insecticidal, immunomodulatory and antiproliferative properties [6,7,8,9]. Limonin is extensively studied for its antioxidant and anti-inflammatory properties and is considered of interest for the treatment of liver diseases and as a cytoprotective agent to protect against organ damage [10,11,12]. However, this compound presents limited bioavailability and can lead to renal and hepatic toxicities [13]. It provides a useful starting material for elaborating rearranged bioactive molecules [14].

**Figure 1:**
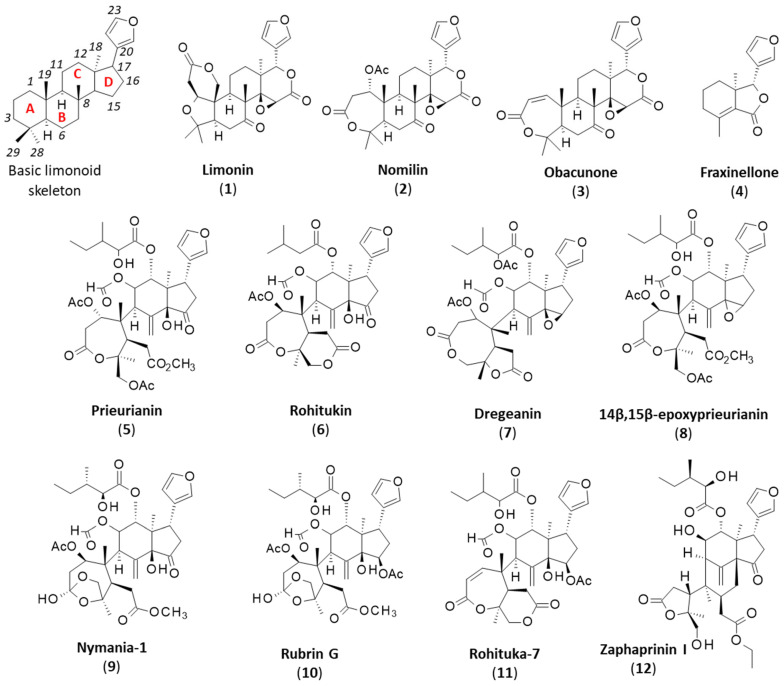
Chemical structures of the basic limonoid skeleton (with the four cycles A-B-C-D and numbering system) and various intact or fragmented (degraded) limonoid cores.

The furanolactone core structure is the signature of limonoids (Figure 1). They are biosynthesized from a 30-carbon precursor (protolimonoid) via a scaffold rearrangement process implicating many enzymes [15]. Complex modifications or remodeling of the initial scaffold can occur [16,17]. The modifications lead to so-called deformed or rearranged limonoids. The modifications include B-ring cleavage reactions, B/C-ring rearrangements and various types of cyclization, altogether leading to many different scaffolds and ring systems [14]. The structural diversity is large among limonoids [18,19].

There are also fragmented limonoids, often called degraded limonoids, corresponding to smaller products with ring openings associated with the loss of a skeletal fragment [20]. In general, these smaller molecules are easier to access by chemical synthesis than complex full limonoids, and as such, they provide starting structures for the total or semi-synthesis of natural products or original derivatives derived from naturally occurring limonoids. Degraded limonoids offer convenient scaffolds for the design of bioactive molecules, such as novel antibacterial agents [21]. This is typically the case for fraxinellone (**4**) isolated from the root bark of *Dictamnus* and *Melia* plants. This compound displays marked insecticidal and anticancer activities, linked to its anti-inflammatory and immuno-modulatory properties [22]. Fraxinellone (**4**) and congeners (e.g., fraxinellonone and isofraxinellone) have led to the synthesis of derivatives and novel insecticide candidates [23,24,25].

Several types of limonoids can be found in Meliaceae plants, with a highly complex structure (e.g., azadirachtin) or a simpler scaffold (such as the cedrelone and azadirone classes). Potent insecticidal agents can be found in each subgroup of compounds [26,27,28]. A less-known subgroup of limonoids is the prieurianin type, which includes three main members: prieurianin (**5**), rohitukin (**6**) and dregeanin (**7**) (Figure 1). The present review provides an analysis of prieurianin-type limonoids to highlight the structural diversity within the family and the origins of these compounds and, especially, to discuss their mechanisms of action. A novel direction is proposed for prieurianin based on the mechanism of the degraded limonoid fraxinellone.

## 2. Prieurianin-Type Limonoids: Structure and Origins

Prieurianin (**5**) is a tetranortriterpenoid that was first isolated from the timber of the tree *Trichilia prieuriana* A. Juss. (synonym: *Trichilia senegalensis* C.DC.) collected in Nigeria, together with many other limonoids [29]. *T. prieuriana* (Meliaceae) is generally a tall tree (up to 30 m) that is well fluted (up to 100 cm in diameter) with a dense crown. It is found in different parts of tropical Africa. The wood is used for the construction of local houses, tool handles and kitchen utensils. All parts of the plant (leaves, bark, twigs and roots) can be used for diverse medicinal usage. For example, a decoction of leafy twigs is taken to treat bronchitis and edema, whereas the pulverized roots are used as a treatment against ascariasis and as purgatives. It is a multi-purpose medicinal tree [30]. *Trichilia* species are commonly used in traditional medicine in Africa, not only *T. prieuriana* but also *T. dregeana* and *T. emetica* [31]. Extracts prepared from these plants are considered active and safe. An ethanolic leaf extract of *T. prieuriana* has not revealed any major toxicity, even when administered to rats at a high dose (LD_50_ > 5000 mg/kg) [32].

**Figure 2:**
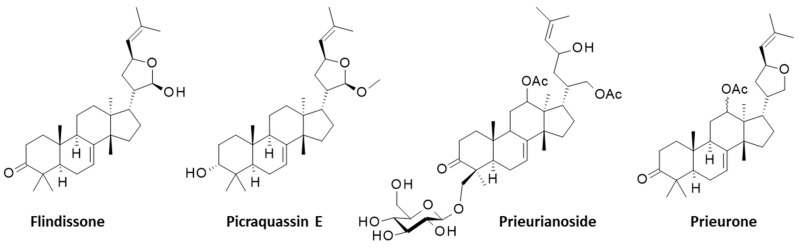
Four other limonoids isolated from *Trichilia prieuriana* A. Juss. together with prieurianin.

Prieurianin was discovered in 1965, but the complex highly oxidized structure of the molecule was elucidated only ten years later based on a precise NMR analysis. It is an A,B-seco-type degraded limonoid derived from the cleavage of the C-3/C-4 and C-7/C-8 bonds of the canonical limonoid framework, with a rearranged new oxo-ring formed by recyclization [33]. Other compounds of interest have been isolated from *T. prieuriana*, notably from the roots of a plant collected in Cameroon (Africa), such as the classical (ring-intact) limonoids flindissone and picraquassin E [34,35]. The protolimonoid glucoside prieurianoside and the limonoid prieurone have also been isolated from the leaves of the same plant [36,37], but these compounds are structurally distinct from prieurianin (**5**) (Figure 2).

**Figure 3:**
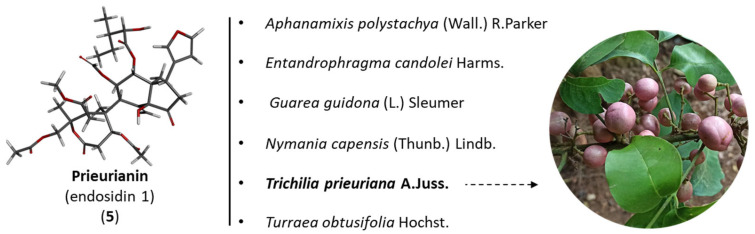
Plants containing prieurianin. An illustration of the leaves, twigs and fruits of *T. prieuriana* is presented (from M. Simo-Droissart, https://identify.plantnet.org/fr/k-world-flora/species/Trichilia%20prieuriana%20A.Juss./data (accessed on 18 February 2024)).

Prieurianin has been found in a few other species, notably the root bark of the medicinal tree *Guarea guidona* (L.) Sleumer (Meliaceae, found in *French Guiana,* South America), together with its analog 14β,15β-epoxyprieurianin (**8**) [38]. Prieurianin has been found in four other Meliaceae: (i) *Turraea obtusifolia* Hochst. [39], (ii) *Nymania capensis* (Thunb.) Lindb. [40], (iii) *Aphanamixis polystachya* (Wall.) R. Parker [41] and (iv) *Entandrophragma candolei* Harms. [42,43] (Figure 3). The bark of this latter African plant was shown to contain the same epoxy–prieurianin derivative (**8**) as in *Guarea guidona* [42]. The bark and timber of *N. capensis* afforded prieurianin (**5**) and the related anti-plasmodial product nymania-1 (**9**) lack the C29-acetate of prieurianin and bear an *ortho* ester (Figure 1) [40,44]. Prieurianin can be isolated from the bark of *A. polystachya* but also from the seeds of the plant, together with rohitukin (**6**) and other rohituka limonoids [45].

**Figure 4:**
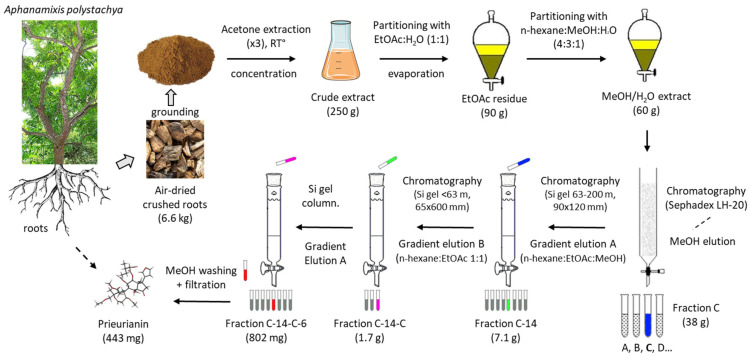
Prieurianin isolation process from the plant *Aphanamixis polystachya* (Wall.) R. Parker, as originally reported in [41]. The successive steps are schematized to show the full extraction/chromatography process, which afforded 443 mg of purified prieurianin starting with 6.6 kg of the dried roots.

An appropriate plant to obtain quantities of prieurianin is the medicinal plant *Aphanamixis polystachya* (Wall.) R. Parker (also known as *Amoora rohituka* (Roxb.) Wight and Arn.) for at least three reasons: First, a specific procedure (depicted in Figure 4) has been described to obtain and purify the compound. The multistep process is long and tedious, with successive solvent extractions and chromatographic steps, but the global yield is satisfactory. The authors previously reported the isolation of 443 mg of (**5**) starting with 6.6 kg of air-dried roots, which corresponds to a correct yield of 67 mg/kg. The process was used to obtain prieurianin (**5**) in addition to triterpenoids such as aphataiwanin A–D and limonoids such as rohituka-3 and -7; nymania-1; and rubrin G [41]. Second, the product can be found in other parts of the plant, including the seeds and fruits, thus providing a renewable source of crude materials [46]. Third, *A. polystachya* is abundant, notably in Bangladesh, where the seed oil (known as pithraj seed oil) is exploited for the production of biodiesel [47,48,49]. Therefore, a phytochemical supply chain could be developed, but a comparison to other sources should also be considered.

The plant *Aphanamixis polystachya* is a prominent source of tetranortriterpenes and limonoids. Several key compounds have been isolated from these plant roots, including prieurianin (**5**) and nymania 1 (**9**) but also the related limonoids rubrin G (**10**) and rohituka-7 (**11**) [41] (Figure 1). Rubrins A-G are degraded limonoids originally from the roots of the tree *Trichilia rubra* C.DC., native to tropical South America [50]. *A. polystachya* contains other prieurianin-type limonoids, such as the insecticidal compounds designated as aphapolynins and aphanamixoids, isolated, respectively, from the fruits and leaves of the plant [46,51]. Recently, other complex compounds, called aphanaonoids, with an oxygen-bridged scaffold, were identified from *A. polystachya*, but no bioactivity was reported [52]. Several other prieurianin-type limonoids have been isolated from the roots, aerial parts, fruits and seeds of various *Trichilia* and *Munronia* species (Table 1). Limonoids are abundant in *Trichilia* species, but prieurianin-type limonoids are not so frequent [53,54]. An interesting series is that of the compounds called zaphaprinins A–Y from *Aphanamixis grandifolia*, with potent insecticidal agents such as zaphaprinins I (**12**), for example [27,55].

**Figure 5:**
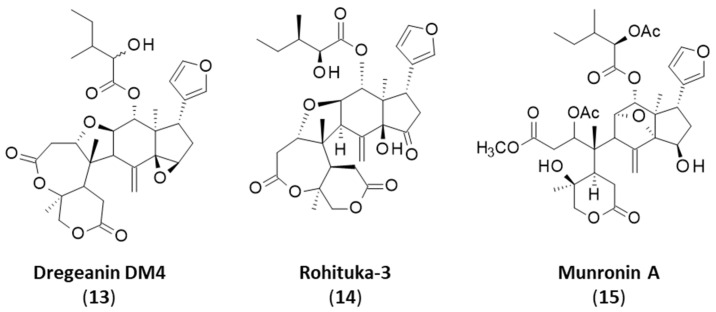
Prieurianin-type limonoids **13**–**15**.

Rohitukin (**6**) and rohituka compounds correspond to a small group of prieurianin-type limonoids found in some Meliaceae, notably in neem (*Azadirachta indica* A. Juss.), a versatile medicinal plant that also contains the classical limonoid nimbolide [74,75,76]. Both prieurianin (**5**) and rohitukin (**6**) have been found in *Turraea obtusifolia* [39]. Rohitukin (**6**) has also been isolated from *Aphanamixis polystacha*, together with dregeanin (**7**) [77,78,79]. The limonoid rohitukin is less active than prieurianin as an insecticidal agent [39]. There is a complete series of related limonoids, designated rohituka-#, such as rohituka-3, -5, -7, -14 and -15, isolated from the seeds of *A. polystacha* [80,81]. They are insecticidal A,B-seco limonoids that are generally much less active than prieurianin [82]. The same observation can be made for compounds related to dregeanin and designated dregeana-#, such as dregeana-1 to dregeana-5 [31]. The limonoid dregeanin has also been isolated from the roots of *Turreanthus africanus* and found to be poorly active as an antibacterial agent [83]. There is also a related compound designated dregeanin DM4 (**13**) from the West African species *Trichilia welwitschia*, characterized as a modest inhibitor of acetylcholinesterase (AChE), a little less active than rohituka-3 (**14**) [84,85] (Figure 5).

Among the 70 or so prieurianin-type limonoids mentioned in Table 1, only a few have shown interesting biological properties, such as the antiproliferative agent munronin A (15), which is active against SW480 colon cancer cells [65]. The objective of the present study is not to detail these 70 compounds; there are recent comprehensive reviews for that [54,86,87]. Our analysis mainly focuses on the pharmacological properties of the lead compound prieurianin (**5**), with a new proposal for a drug target. Most studies on these prieurianin-type limonoids are concerned with the structural characterization of new complex molecular entities, with only preliminary biological tests (generally one or two specific cellular or biological assays). The pharmacological potential of these natural products remains little known. However, a recent discovery made with fraxinellone (**4**) led us to propose a new mechanistic option for prieurianin (**5**).

## 3. Bioactivities of Prieurianin and Analogs

Prieurianin exerts marked insecticidal action. The compound has been shown to antagonize molting steroid hormone 20-hydroxyecdysone activity in *Drosophila* cells. Prieurianin is significantly more potent than rohitukin as an antagonist of 20-hydroxyecdysone action in *Drosophila melanogaster* BII cells (ED_50_ = 10 µM and 125 µM) [39]. Moreover, antifeedant activity has been reported when using the pod borer *Helicoverpa armigera* (Hubner) (Lepidoptera: Noctuidae). Prieurianin and its epoxy derivative reduce the feeding of larvae without inducing cytotoxic effects [42,51].

In addition, prieurianin has revealed both antiadipogenic and anorexigenic effects in mice. The compound inhibits the proliferation and differentiation of preadipocytes into adipocytes and modifies mature adipocytes, inducing their dedifferentiation or delipidation. These effects lead to significant weight loss by reducing energy intake in obese mice [88]. In one study, when mice under a high-fat diet received prieurianin at 1–3 mg/kg for 3 weeks (intraperitoneally), a dose-dependent loss of weight was observed. The mice recovered a normal weight at the end of the treatment period, and the weight loss was accompanied by a major (70–80%) decrease in food consumption. Prieurianin exhibited marked anti-obesity properties [88]. Surprisingly, this interesting activity has not been investigated further, perhaps due to the difficulty in accessing the compound. However, similar effects have been reported with a few other limonoids, notably with nimbolide, which suppressed high-fat-diet-induced obesity in rats in [89]. Nomilin also displays anti-obesity effects [90,91]. The antiadipogenic and anorexigenic properties of prieurianin merit further studies. There is a constant need for efficient and safe products to treat patients with overweight or obesity.

Antiproliferative activity has been reported in prieurianin. The compound has been shown to inhibit the proliferation of KB3-1 human cervix carcinoma cells (IC_50_ = 1.47 μM). This effect has been attributed to the potential binding of the compound to molecular targets such as α,β-tubulin dimer, DNA-topoisomerase I and human neutrophil collagenase (MMP-8), but these predicted interactions (based on molecular modeling) have not yet been validated experimentally [43]. Moreover, the marked antiproliferative activity reported with KB3-1 cells is a little surprising because another study concluded that prieurianin exerts no cytotoxic action against Hep-G2 (human hepatocellular carcinoma), A549 (human lung carcinoma) or MCF-7 (human breast carcinoma) [IC_50_ > 40 µg/mL) and modest activity against HEp-2 cells (laryngeal cancer cells) (IC_50_ = 16.8 µg/mL (22 μM)) [41]. It is likely that the mechanism is multi-factorial and dependent on the cell species (cancer cells or insects) and the histological type. More work is needed to clarify this cytotoxicity aspect, including integrative approaches and biology-based screening methods to further delineate the potency and mechanism of action of prieurianin, as achieved with other limonoids [92,93].

## 4. Potential Molecular Targets of Prieurianin and Analogs

**Figure 6:**
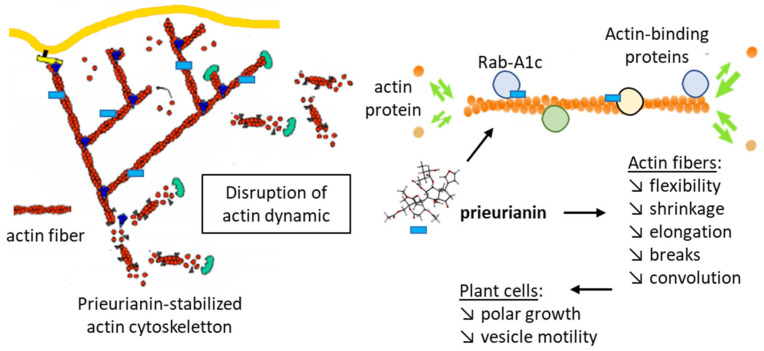
Effects of prieurianin on actin dynamics. Prieurianin stabilizes the actin cytoskeleton, reducing actin fiber flexibility and shrinkage and causing changes in vesicle trafficking. The drug action implicates actin-binding proteins and the modulation of endosome trafficking [95]. The action of prieurianin (blue rectangle) is schematized to illustrate binding to actin-binding proteins (green and yellow circles), including Rab-A1c GTPase (blue circles) and the resulting effects in actin fibers and plant cells.

Image-based screening for chemicals capable of modulating the trafficking of proteins to the plasma membrane via endosomes in plants has led to the identification of a compound called endosidin 1. This compound was found to block the endocytosis of auxin transporter proteins in the roots of the plant *Arabidopsis thaliana* (L.) Heynh. Endosidin 1 affects endosome trafficking at the stage of early endosome formation. The compound changes the distribution of markers residing in the trans-Golgi network/early endosomes [94]. A subsequent study revealed that endosidin 1 is in fact prieurianin and functions as a modulator of actin cytoskeleton dynamics. Moreover, prieurianin was identified as an effector of the circadian clock in *A. thaliana*, causing a shortening of circadian period lengths. The authors stated that prieurianin affects the actin cytoskeleton through a mode of action distinct from that of previously described inhibitors of actin dynamics. It does not function as a stabilizer or destabilizer of actin filaments but most likely targets an actin-associated protein implicated in cytoskeleton formation and vesicle trafficking [95]. The compound impairs actin dynamics via the indirect stabilization of the actin cytoskeleton (Figure 6). The effects of the natural products have been evidenced using both plant cells (hypocotyl cells from *Arabidopsis thaliana*) and mammalian cells (BSC-1 monkey epithelial fibroblasts). Prieurianin severely alters the architecture of the actin network, reducing filament flexibility and shrinkage and decreasing the number of breaks per filament, but the filament growth rate is not affected. The compound has shown an atypical effect on the dynamics of the actin cytoskeleton, reducing actin rearrangements [96]. As such, the mechanism of action of prieurianin seems to be distinct from that of azadirachtin A, the major limonoid found in neem (*Azadirachta indica*), which induces depolymerization of actin [96]. Computational analyses have helped to define the actin-binding site of azadirachtin A, located in subdomain 4 of a subunit (n + 2) of actin [97,98]. Prieurianin functions differently, altering membrane trafficking from the trans-Golgi network via actin-binding proteins implicated in transport processes from the trans-Golgi network, such as Rab GTPases and, in particular, a member of the Rab-A1 subclass called Rab-A1c [99,100]. These Rab proteins are key regulators of membrane transport in eukaryotes [101].

**Figure 7:**
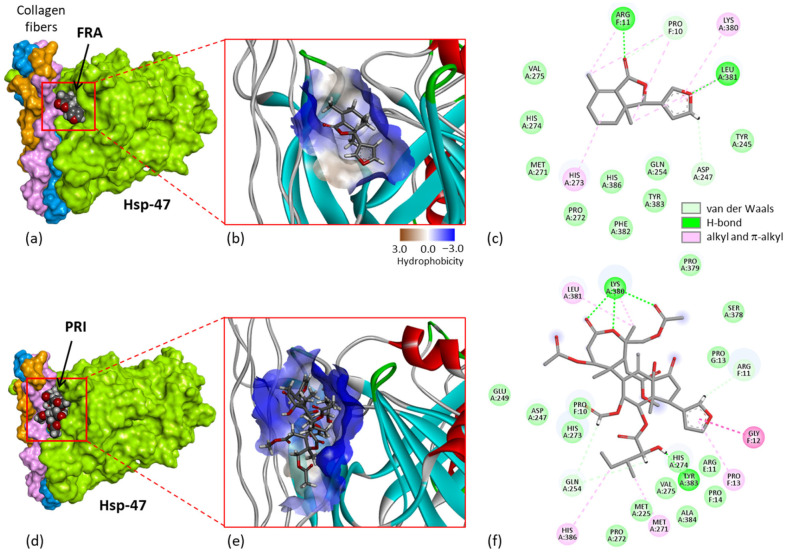
Molecular models of fraxinellone (**a**–**c**) and prieurianin (**d**–**f**) bound to Hsp47 (protein data bank (PDB) code 3ZHA). (**a**) Surface model with a close-up view of the binding cavity that accommodates the compound (**4**). (**b**) A view of the fraxinellone-binding site, with the hydrophobicity surface surrounding the drug-binding zone (color code indicated). (**c**) Binding map contacts for (**4**) bound to Hsp47 (color code indicated). Same models in panels (**d**–**f**) for compound (**5**). The modeling analysis was performed as previously described in [109,110].

A recent study performed with the degraded limonoid fraxinellone (**4**) brought other key information to help understand the mechanism of action of these limonoids. Fraxinellone is an insecticidal and anticancer limonoid isolated from the root bark of *Dictamnus dasycarpus* [22]. The compound has been shown to interact with heat shock protein 47 (Hsp47, also known as Serpin H1), which is implicated in the development of intestinal fibrosis. A direct binding of (**4**) to the purified protein has been evidenced by surface plasmon resonance (Kd = 3.542 × 10^−5^ M), and a modeling analysis helped to define the location of the binding site with the implication of some key amino acids in the binding process, notably residues Tyr383 and Asp385. This drug interaction inhibits and destroys the complex between the chaperone Hsp47 and collagen, thereby perturbating procollagen folding and collagen processing [102]. The interaction with Hsp47 directly implicates the furan unit of fraxinellone. Fraxinellone can bind reasonably well to Hsp47, forming stable protein complexes at the interface of the Hsp47-collagen region, as represented in Figure 7a–c. There is a protein cavity at the junction between the three collagen fibers and the protein, accessible for drug binding.

The regulation of Hsp47-collagen’s interaction with fraxinellone accounts for the antifibrotic action of the limonoid [102], and it may also contribute to its antimetastatic activity because Hsp47 is a known stimulator of metastasis in solid tumors, notably in breast cancer [103]. These observations prompted us to consider that, by analogy, prieurianin could bind to Hsp47 via its fraxinellone-like moiety. The tetrahydrobenzofuranone unit with the appended furanyl group in fraxinellone is similar to the core of prieurianin, with different substitutions. Prieurianin is significantly larger than fraxinellone, with an appended 7-oxo-oxepanyl ring, but its binding to Hsp47 is apparently conceivable, as inferred from a preliminary molecular modeling analysis (Figure 7d–f). The docking analysis suggested that prieurianin can form much stabler complexes with Hsp47 compared with fraxinellone. Both the calculated empirical energy of interaction (ΔE) and free energy of hydration (ΔG) are much more favorable with prieurianin than with fraxinellone. The ΔE value calculated with prieurianin (**5**) is 2.5-fold more negative than that measured with fraxinellone (**4**) (−41.7 kcal/mol vs. −106.5 kcal/mol for **4** and **5**, respectively) (Table 2). The difference is considerable and suggests that prieurianin exhibits a high affinity for this binding site on Hsp47. Multiple drug–protein contacts stabilize the complex, including H-bonds with the key residue Tyr383 and with residue Lys380 in contact with the oxepanyl ring of prieurianin, together with multiple weaker hydrophobic interactions (Figure 7f). Interestingly, the same trend was observed with the related products dregeanin and rohitukin. The ΔE values for the three compounds rank in the order prieurianin < dregeanin < rohitukin (Table 2). The modeling analysis strongly supports the potential binding of prieurianin to the same site of Hsp47 as the fragmented limonoid fraxinellone. For the time being, this is only a computer-based prediction, with the inherent inaccuracies of molecular docking [104]. The limited reliability of scoring functions is known [105], but the hypothesis is entirely plausible considering the capacity of other limonoids to function as heat shock protein inhibitors, such as gedunin and chisomicine D [106,107,108].

The interaction of prieurianin with Hsp47 would be entirely compatible with its insecticidal effects. Different chaperone proteins have been shown to play a role in the signaling of the ecdysone receptor, which is a major steroid receptor in insects [112,113,114]. Heat shock proteins play important roles in the nucleocytoplasmic shuttle of the ecdysone receptor. Notably, 20-hydroxyecdysone regulates the expression of the chaperone Hsp70 and other small Hsp proteins [115]. The expression of heat shock proteins and the development of the smooth endoplasmic reticulum have been observed in some insects, such as the armyworm *Spodoptera eridania* and the predator *Ceraeochrysa claveri*, in response to cellular damage [116]. Limonoids could block the expression and/or function of Hsp. For example, Hsp23 is considered to be a potential target of the neem limonoid azadirachtin in *Drosophila melanogaster* larvae [117]. The two andirobin-type limonoids moluccensin-N and moluccensin-O have been shown to interact with Hsp90 [118]. It is known that Hsp proteins play a role in insect metamorphosis and that stress induces the expression of heat shock protein genes [119,120].

## 5. Conclusions and Perspectives

This work shed light on a group of degraded limonoids not frequently studied. Prieurianin is the leader molecule in a series that includes about 70 compounds (Table 1). Most of these compounds have been structurally described, together with corresponding isolation processes from different plant species. However, their pharmacological activities and mechanisms have rarely been investigated. Priority is generally given to the most abundant polycyclic limonoids in plants, in particular, the classical intact limonoids, such as limonin and nomilin, and to smaller fragmented limonoids with easier synthetic access. The field of limonoids is very active, with more than 1600 compounds characterized over the past 15 years [2] and a profusion of synthetic derivatives (>800) elaborated during roughly the same period [121]. But in this vast molecular armamentarium, the use of degraded limonoids of the prieurianin type has been little considered, either because the molecules are too complex structurally or not well known and hardly available or because of the limited information concerning their bioactivities and mechanism of action. It is time to highlight the potential benefits of prieurianin, which is an insecticidal agent with an atypical capacity to modulate the dynamics of the actin cytoskeleton in cells. In recent years, novel bioactive prieurianin-type limonoids have been identified, such as trichilianones A-E [72] and munronins T-U [68]. The latter example is appealing because the mode of action invoked for these two compounds is an inhibition of the expression of the two Hsp genes *NtHsp70*–*1* and *Nthsp70*–*261*. There is probably a strong link between the impact of the compounds on the Hsp machinery and their antiviral effects. Further investigation into the mechanism of action of prieurianin and related compounds is warranted. Prieurianin is a convenient tool for studying interference with endocytosis and vesicular recycling in plant cells [122,123]. This natural product warrants better consideration as an insecticidal agent and as an antiadipogenic molecule. The hypothesis that prieurianin binds well to the collagen-binding protein Hsp47 is attractive and opens new perspectives for the design of other compounds with antifibrotic properties because this protein is a major recognized target to combat pulmonary fibrosis and other fibrotic diseases [124,125,126]. Hsp47 inhibitors mediate antifibrotic effects by suppressing the overexpression of collagen and inhibiting the viability and migration of fibroblasts [127]. Both fraxinellone and prieurianin may provide a molecular basis for the development of novel antifibrotic therapeutics.

## Figures and Tables

**Table 1:** Other prieurianin-type limonoids found in Meliaceae and their properties.

Compounds	Plants ^1^	Plant Parts Used	Reported Activities	References
Aphanamixinin	*A. polystachya*	Bark	No activity reported.	[56,57]
Aphanamixoids A–B	*A. polystachya*	Leaves and twigs	Moderate antifeedant activity against the beet armyworm (*Spodoptera exigua*).	[58]
Aphanamixoids K–P	*A. polystachya*	Leaves and twigs	Weak antifeedants against the lepidopteran agricultural pest *Helicoverpa armigera*.	[51]
Aphanaonoids A–H Aphanaonoids I–J	*A. polystachya* *A. sinensis*	Leaves and twigs	No activity reported.	[52]
Aphapolynins A–B	*A. polystachya*	Fruits	Modest antiproliferative activity of aphapolynin A against two carcinoma cell lines.	[59]
Aphapolynins C–I	*A. polystachya*	Fruits	Aphapolynin C displayed moderate activity against the phytopathogenic fungus *Pythium dissimile* and insecticidal effects.	[46]
Ciparasin P	*C. cinerascens*	Leaves	Significant anti-HIV activity and little cytotoxicity against MT-4 cells.	[60]
Dysoxylumin A	*D. mollissimum*	Twigs	Cytotoxic activity against A549 cancer cell.	[61]
Dysoxylumins A–C	*D. hainanense*	Bark	No activity reported.	[62]
Monbasone, monbasol	*T. mombasana*	Roots	No activity reported.	[63]
Munronoid O	*M. unifoliolata*	Whole plant	Inhibition of TMV infection.	[64]
Muronin A	*M. henryi*	Twigs	Cytotoxic activity against cancer cell lines.	[65]
Muronin O, P, Q	*M. henryi*	Twigs	Antiviral effect against the tobacco mosaic virus (TMV).	[66,67]
Munronin T, U	*M. henryi*	Twigs	Weak protection from tobacco mosaic virus (TMV) infection.	[68]
Munropins A–F	*M. pinnata*	Aerial parts	No cytotoxic activity.	[69]
Trichavensin	*T. havanensis*	Seeds	No activity reported.	[70]
Trichilianones A–D	*T. adolfi*	Bark	Weak antiparasitic activity against *Leishmania braziliensis* promastigotes in vitro.	[71]
Trichilones A–E	*T. adolfi*	Bark	Weak cytotoxic activity.	[72]
Trichirokin	*T. emetica*	Stem bark	No antibacterial or cytotoxic activity.	[73]
Zaphaprinins A–Y	*A. grandifolia*	Fruits	Marked insecticidal activity of zaphaprinins I and R against the grain aphid *Sitobion avenae* and the diamondback moth *Plutella xylostella*.	[55]

^1^*A. grandifolia*: *Aphanamixis grandifolia* Blume; *A. polystachya*: *Aphanamixis polystachya* (Wall.) R. Parker; *A. sinensis*: *Aphanamixis sinensis* F.C.How and T.C.Chen.; *C. cinerascens*: *Cipadessa cinerascens* (Pellegr) Hand.-Mazz.; *D. hainanense*: *Dysoxylum hainanense* Merr.; *D. mollissimum*: *Dysoxylum mollissimum* var. *glaberrimum*. (F.C.How and T.C.Chen) P.Y.Chen; *M. henryi*: *Munronia henryi* Harms; *M. pinnata*: *Munronia pinnata* (Wall.) W. Theob.; *M. unifoliolata*: *Munronia unifoliolata* Oliv.; *T. adolfi*: *Trichilia adolfi* Harms; *T. emetica*: *Trichilia emetica* Vahl; *T. havanensis*: *Trichilia havanensis* Jacq; *T. mombasana*: *Turraea mombasana* Hiern ex C.DC.

**Table 2:** Calculated potential energy of interaction (ΔE) and free energy of hydration (ΔG) for the interaction of selected limonoids with heat shock protein 47 (Hsp47) ^1^.

Compounds	CID *	ΔE (kcal/mol)	ΔG (kcal/mol)
Fraxinellone	124039	−41.70	−19.80
Prieurianin	329486	−106.50	−30.60
Rohitukin	99982	−88.50	−34.45
Dregeanin	433157	−92.25	−19.75

^1^ The docking process has been described previously [109,110]. The docking analysis was performed with Hsp47 (PDB code 3ZHA [111]), keeping the following amino acids totally flexible: Arg222, Tyr245, Asp247, Met271, His273, Leu381, Tyr383, Asp385, His386 and Arg11 (E collagen unit). * CID: Compound identity number from the PubChem database.

## Data Availability

The data are contained within the article.
